# Progression to Dementia in Mild Cognitive Impairment With Lewy Bodies or Alzheimer Disease

**DOI:** 10.1212/WNL.0000000000012024

**Published:** 2021-06-01

**Authors:** Calum A. Hamilton, Fiona E. Matthews, Paul C. Donaghy, John-Paul Taylor, John T. O'Brien, Nicola Barnett, Kirsty Olsen, Rory Durcan, Gemma Roberts, Joanna Ciafone, Sally A.H. Barker, Michael Firbank, Ian G. McKeith, Alan J. Thomas

**Affiliations:** From the Translational and Clinical Research Institute (C.A.H., P.C.D., J.-P.T., N.B., K.O., R.D., G.R., J.C., S.A.H.B., M.F., I.G.M., A.J.T.) and Population Health Sciences Institute (F.E.M.), Newcastle University; and Department of Psychiatry (J.T.O.), University of Cambridge School of Clinical Medicine, UK.

## Abstract

**Objective:**

To determine whether mild cognitive impairment with Lewy bodies or mild cognitive impairment with Alzheimer disease differ in their rates of clinical progression to dementia, we undertook longitudinal observation of mild cognitive impairment cases with detailed clinical assessment of Lewy body diagnostic characteristics.

**Methods:**

Two prospective longitudinal cohorts including 111 individuals ≥60 years of age with mild cognitive impairment were assessed annually to track cognitive and clinical progression, including the presence or absence of core clinical features and proposed biomarkers of dementia with Lewy bodies. Multistate modeling was used to assess the associations of diagnostic characteristics of dementia with Lewy bodies with clinical progression from mild cognitive impairment to dementia, with death as a competing outcome.

**Results:**

After a mean follow-up of 2.2 years (range 1–6.7 years), 38 of the 111 (34%) participants progressed to dementia: 10 with AD, 3 with possible dementia with Lewy bodies, and 25 with probable dementia with Lewy bodies. The presence of any Lewy body disease characteristic was associated with an increased hazard of transition to dementia; this risk further increased as more diagnostic characteristics were observed (hazard ratio 1.33 per characteristic, 95% confidence interval [CI] 1.11–1.60) and was especially high for those experiencing complex visual hallucinations (hazard ratio 1.98, 95% CI 0.92–4.29) or cognitive fluctuations (hazard ratio 3.99, 95% CI 2.03–7.84).

**Conclusions:**

Diagnostic characteristics of Lewy body disease are associated with an increased risk of transition from mild cognitive impairment to dementia.

Dementia with Lewy bodies (DLB) has a worse prognosis than Alzheimer disease (AD), with increased hospitalization^1^ and shorter survival time.^2^ We have previously found^3^ that the cognitive prodrome of DLB, mild cognitive impairment (MCI) with Lewy bodies (MCI-LB), for which research diagnostic criteria were recently published,^4^ was more likely to demonstrate a progressive cognitive decline than MCI due to AD (MCI-AD). The respective cognitive prodromes may therefore progress at different rates, as in dementia. However, it remains unclear as to whether an MCI with core clinical features of DLB (REM sleep behavior disorder [RBD], parkinsonism, complex visual hallucinations, and cognitive fluctuations) has a worse clinical prognosis with faster dementia onset than MCI-AD and whether progression risk differs between those with different clinical features.

RBD and Parkinson disease are risk factors for neurodegenerative disease and dementia but may remain as isolated diagnoses without cognitive decline for many years^5,6^ and may not manifest in greater risk over the short term of this study. Psychiatric symptoms in an amnestic MCI are associated with faster conversion to dementia^7^ and faster decline in AD,^8,9^ so MCI with core neuropsychiatric symptoms of DLB (visual hallucinations or cognitive fluctuations) may progress to dementia faster than MCI without these features.

We hypothesized that MCI-LB would have a greater annual risk of clinical transition to dementia than MCI-AD; specific characteristics of DLB would confer differing risks of dementia onset; and cognitive fluctuations and visual hallucinations would be associated with increased dementia risk, whereas RBD and parkinsonism would not.

## Methods

### Participants

Participants were included from 2 prospective longitudinal cohorts, for which recruitment has been described previously in detail,^10,11^ with the second cohort differing only in its inclusion of an additional indicative biomarker for MCI-LB: 123I-metaiodobenzylguanidine (MIBG) cardiac scintigraphy. Prospective participants were recruited from local memory and secondary care psychiatric services, older people's medical services, and neurology and specialist Lewy body clinics in Northeast England, where they had received a recent health service diagnosis of MCI and had either any core feature of Lewy body disease or other supportive symptoms associated with the presence of Lewy body diseases but also found in AD, for example, a history of falls, general sleep disturbance, or hyposmia. Exclusion criteria were the presence of a possible frontotemporal or vascular etiology (when clinical features suggested that the patient might fit the possible behavioral variant frontotemporal dementia clinical criteria, or evidence of clinical stroke in clinical notes or MRI) at either baseline or after follow-up, parkinsonism predating onset of cognitive symptoms by >1 year, and either dementia or absence of objective cognitive impairment. Inclusion criteria were age ≥60 years and a diagnosis of MCI according to National Institute on Aging–Alzheimer’s Association (NIA-AA) criteria,^12^ that is, subjective and objective evidence of cognitive decline with retention of independent function and therefore not meeting clinical criteria for dementia.^13,14^ Participants from both cohorts were selected for inclusion when they had at least 2 observations available or had died before the second observation. A self-selected subset of participants participated in both studies; these are included within the LewyPro cohort, but had additional imaging results are available from the SUPErB cohort (see below).

### Measures and Diagnoses

Participants underwent semistructured interview and neurologic examination with a board-certified medical doctor at baseline and at annual r-assessment. When available, an informant was also interviewed to provide additional information. Clinical notes from these assessments were independently reviewed by a panel of experienced old-age psychiatrists (A.J.T., P.C.D., J.P.T.) who confirmed the clinical diagnosis as MCI according to NIA-AA criteria.^12^ The same panel independently rated the presence or absence of each of the 4 core diagnostic features of DLB.^14^ Assessments of core diagnostic features of DLB (complex visual hallucinations, cognitive fluctuations, RBD, and parkinsonism) were guided by standardized scales: the Clinician Assessment of Fluctuations and Dementia Cognitive Fluctuations Scale, the North-East Visual Hallucinations Inventory, the Mayo Sleep Questionnaire, and the Revised Unified Parkinson's Disease Rating Scale Motor Subscale. However, the presence/absence of core clinical features was a clinical judgment with these scales and all available information from the health service and research records (including neurologic examination) rather than cutoff scores alone. Clinical review was repeated annually, including reassessment of diagnosis (MCI or dementia), with repeated rating of the presence or emergence of any core symptoms of DLB.

Dopaminergic ^123^I-*N*-fluoropropyl-2β-carbomethoxy-3β-(4-iodophenyl) single-photon emission CT (FP-CIT) imaging was undertaken as detailed previously,^15^ with images independently rated as normal or abnormal by an experienced trained panel blinded to clinical information. Those included from the second study (n = 64 total, which included 23 from the first study) also underwent MIBG scintigraphy to assess cardiac denervation as an additional biomarker for prodromal DLB. Delayed images were processed blinded to clinical information and quantified to provide a heart:mediastinum uptake ratio, and a cutoff for abnormality was derived from locally recruited cognitively healthy older adults: heart:mediastinum uptake ratio of ≥2 SDs below the healthy mean were rated as abnormal. Symptomatic heart failure was cause for exclusion to prevent diagnostic false positives. The results of both imaging modalities were included in diagnoses.

Diagnosis of MCI-LB was operationalized as outlined in the current consensus research criteria^4^ for diagnosis of prodromal DLB. Cases with MCI, no DLB core clinical features or indicative biomarkers, and no features of other potential causes of dementia, for example, vascular or frontotemporal etiology, were diagnosed as having MCI due to AD (MCI-AD), in accordance with NIA-AA criteria.^12^ MCI cases with either any 1 core DLB clinical feature and no indicative biomarker or no core clinical features but an indicative biomarker present were diagnosed as having possible MCI-LB. Individuals with MCI and either ≥2 DLB core clinical features or 1 core clinical feature and an indicative biomarker received a diagnosis of probable MCI-LB. For direct comparison in the multistate modeling analysis, all MCI subgroups were included as a single MCI group, with their clinical characteristics as covariates.

When judged to meet NIA-AA criteria for all-cause dementia,^13^ participants received a diagnosis of dementia and ended involvement in the study. Diagnosis of all-cause dementia was based on reported loss of independent function along with evidence of cognitive decline as judged by the panel. Core symptom presence and abnormal biomarkers were subsequently assessed as above, and their final dementia diagnosis was rated according to current consensus clinical criteria for DLB or AD.^13,14^

Cognitive assessments were undertaken with a detailed panel of neuropsychological tests administered separately from the clinical interview, with a median of 12 days between these assessments. In-depth cognitive profiles of this cohort have been detailed previously^10^: the Addenbrooke's Cognitive Examination–Revised provided global cognitive scores reported here, and a derived Mini-Mental State Examination score contextualized the mental status of this cohort on study entry. Cognitive profile was not incorporated into differential diagnosis, which was based on clinical assessment only. Instrumental activities of daily living were recorded, and the Movement Disorder Society Unified Parkinson's Disease Rating Scale–Part III: Motor Examination was administered to quantify motor impairments; quantified scores of these were included for research purposes, but review of functional impairment and parkinsonism was based on clinical reasoning, not score cutoffs.

Socioeconomic background was anticipated to be a confounding variable; local community deprivation has been directly associated with cognitive dysfunction in older age and indirectly via other deprivation-related factors associated with increased risk of conversion to dementia from MCI.^16^ English indices of multiple deprivation deciles were therefore derived from publicly available national statistics^17^ according to each participant's home address at the time of study enrollment. Neighborhood deprivation scores are nationally ranked, and these were sorted into deciles; decile rank of 1 as presented here corresponds to the 10% most deprived neighborhoods in England, and a rank of 10 is among the 10% least deprived neighborhoods.

### Analysis

A competing-risks multistate model was assembled with the msm package for R software (R Institute for Statistical Computing, Vienna, Austria).^18^ This approach provides a flexible framework in which to undertake survival analysis with multiple states (2 competing end-state risks in this case but may be extended to include more complex transition structures) and time-varying covariates (e.g., emergence of clinical features). Three states were defined: MCI, dementia, and death. Dementia and death were treated as competing absorbing states with no subsequent transitions allowed; because participants ceased involvement in the study after conversion to dementia, no further information was available after clinical conversion. Exact dates were recorded for all deaths. Observation times were recorded as a continuous variable (i.e., days since individual baseline assessment/365) to account for any variability in follow-up schedule, with the zero point at the participant's first enrollment in the respective cohort.

All MCI diagnoses were included under the same MCI state. Likewise, all dementia diagnoses were included within a single dementia state. At each observation, participants could either remain as MCI, with or without some change in any covariates (see below), or progress to dementia or death. The emergence of Lewy body disease characteristics later in the MCI course and their association with subsequent dementia transitions could therefore be assessed in this model in a flexible manner.

Covariates theorized to have an association with clinical conversion were included to assess the association of DLB features and other demographic variables with risk of death or dementia: age, deprivation, sex, education (all previous time invariant, assessed at baseline), and number of DLB diagnostic characteristics (time varying). An additional analysis included the same, with each of 6 specific characteristics included as individually present or absent (time-varying except for MIBG imaging): complex visual hallucinations, cognitive fluctuations, parkinsonism, RBD, and FP-CIT and MIBG abnormalities.

Model fit was assessed by Akaike information criterion (AIC) with a lowered AIC value indicating better model fit, with a penalty for inclusion of additional parameters. Covariates were chosen by forward selection leading to the best-fitting models reported here. In the event that a covariate did not improve model fit, it was excluded to favor parsimony. Because MIBG imaging was available for only a subset of the sample, a sensitivity analysis was conducted including only those with this imaging available (either normal or abnormal) to assess whether including this improved model fit. Interaction effects were considered for retained main effects, assessed with the same criteria (model improvements observed with AIC).

### Standard Protocol Approvals, Registrations, and Patient Consents

Ethics approval was given by the National Research Ethics Service Committee North East–Newcastle and North Tyneside 2 (Research Ethics Committee No. 12/NE/0290 and 15/NE/0420), and written informed consent was obtained from all participants.

### Data Availability

Data supporting this analysis are available by request through the Medical Research Council Dementias Platform UK, study references LewyPro and SUPErB.

## Results

### Demographics and Baseline

**Figure 1:**
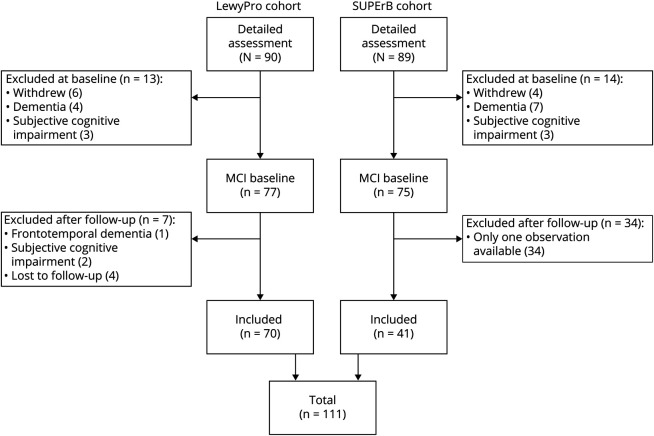
Inclusion and Exclusion of Participants in Cohorts Used for This Analysis MCI = mild cognitive impairment. SUPErB = 123I-MIGB Scintigraphy Utility as a Biomarker for Prodromal Dementia With Lewy Bodies.

One hundred eleven participants were suitable for inclusion (figure 1). Baseline demographic and clinical information is summarized for the overall MCI group and differential diagnostic subgroups in table 1. The mean follow-up time was 2.2 years (SD 1.39 years, median 2.05 years) from baseline, with a range of 1 to 6.7 years of follow-up from baseline. Participants had a median of 3 observations each. While all met criteria for MCI at baseline, those with probable MCI-LB had slightly greater daily functional impairment; instrumental activities of daily living scores were not significantly correlated with global cognitive function (Pearson *r*[98] = 0.16, *p* = 0.11) but had a weak negative association with motor impairment (r[98] = −0.29, *p* = 0.003). Despite comparable cognitive function, patients with probable MCI-LB were more likely to be in receipt of cholinesterase inhibitors at baseline, consistent with local use and recent statements supporting these in the treatment of neuropsychiatric symptoms of Lewy body disease.^19^

**Table 1:**
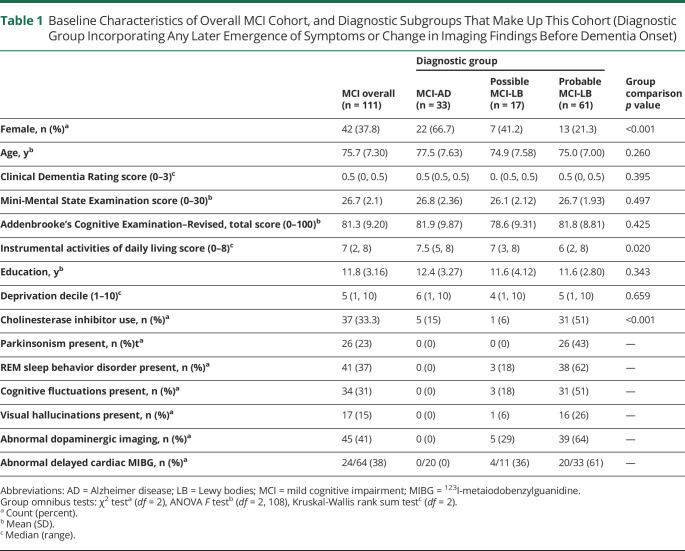
Baseline Characteristics of Overall MCI Cohort, and Diagnostic Subgroups That Make Up This Cohort (Diagnostic Group Incorporating Any Later Emergence of Symptoms or Change in Imaging Findings Before Dementia Onset)

### Dementia Diagnoses and Deaths

Thirty-eight participants (34%) had progressed to dementia and an additional 7 had died (6%) at the time of analysis. Ten had a diagnosis of AD; all 10 had previously been diagnosed with MCI-AD (30% of MCI-AD cases developed dementia). Three had a diagnosis of possible DLB; all had previously been diagnosed as possible MCI-LB (18% of possible MCI-LB cases developed dementia). Twenty-five cases met criteria for a diagnosis of probable DLB; all were previously diagnosed as probable MCI-LB (41% of probable MCI-LB cases developed dementia). Two participants with MCI-AD (6%), 1 with possible MCI-LB (6%), and 4 with probable MCI-LB (7%) had died with a last recorded diagnosis as MCI. Despite similar incidence of death or dementia in the 2 broad groups (total of 36% death or dementia in MCI-AD, 42% in possible or probable MCI-LB), the multistate models indicated that time of onset of these varied by diagnostic characteristics.

### Overall DLB Feature Count

The best-fitting model included age and DLB core feature or indicative biomarker count as main effect covariates, without interactions (table 2). Higher age was associated with an increased risk of death and a small nonsignificant increase in dementia per year. Compared to MCI-AD, each DLB clinical feature or biomarker observed conferred a linearly increasing yearly risk of transition to dementia or death. An increasingly Lewy body–like clinical profile in MCI was therefore associated with worse prognosis as evidenced by an increased annual risk of conversion to dementia or death.

**Table 2:**
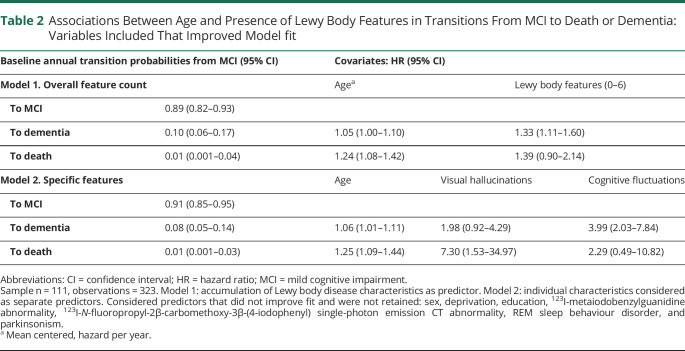
Associations Between Age and Presence of Lewy Body Features in Transitions From MCI to Death or Dementia: Variables Included That Improved Model fit

### Specific DLB Features

The best fitting model favored inclusion of age, visual hallucinations, and cognitive fluctuations as covariates (table 2), without interactions. Increased age was associated with an increased hazard of both dementia and death. The presence of visual hallucinations was associated with increased hazard of death and nonsignificant increased risk of dementia, while cognitive fluctuations were associated with a significantly increased hazard of dementia but not of death. Inclusion of parkinsonism, RBD, or abnormal FP-CIT or MIBG imaging did not improve model fit and thus may not be associated with an increased transition risk to dementia or death compared to MCI-AD.

### Supplementary Analysis: Death or Dementia and Cholinesterase Inhibitor Use

**Figure 2:**
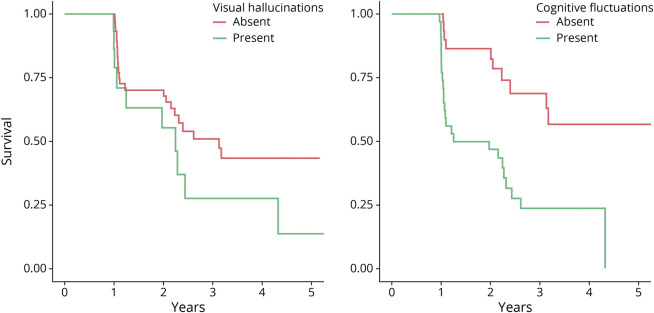
Survival Curves in Mild Cognitive Impairment With Visual Hallucinations or Cognitive Fluctuations at Baseline

Given the low death rates, a supplementary analysis was undertaken incorporating death and dementia into a single end-state outcome to provide a more precise estimate of the general prognosis in MCI. This analysis provided agreement with the main analysis that the presence of DLB clinical features, specifically cognitive fluctuations and visual hallucinations, was associated with increased risk of progression from MCI to a more severe clinical state (table 3). This simple signal is illustrated for each risk factor in figure 2.

**Table 3:**
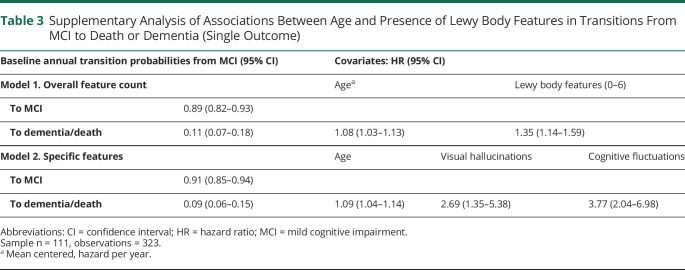
Supplementary Analysis of Associations Between Age and Presence of Lewy Body Features in Transitions From MCI to Death or Dementia (Single Outcome)

In an additional analysis controlling for cholinesterase inhibitor use, the associations between cognitive fluctuations and dementia onset (hazard ratio [HR] 2.6, 95% confidence interval [CI] 1.3–5.0) and between visual hallucinations and death (HR 15.2, 95% CI 2.7–84.7) remained. Visual hallucinations were not clearly associated with dementia risks (HR 1.3, 95% CI 0.5–3.0), but this comparison is limited by a high degree of collinearity between visual hallucination presence and cholinesterase inhibitor use (only 3 of 17 visual hallucinators not using cholinesterase inhibitors at baseline).

## Discussion

We found, as hypothesized, that the presence of DLB characteristics in MCI was associated with a greater annual risk of dementia onset, with death as a competing risk; this risk increased as more characteristics were observed. Cognitive fluctuations in particular were associated with faster dementia onset, while RBD and parkinsonism were not. Visual hallucinations were associated with increased risk of death and with higher but nonsignificant transition to dementia. Parkinsonism and RBD were not individually associated with increased risk of dementia compared to not having these symptoms, nor were dopaminergic and cardiac sympathetic imaging. The data therefore support the hypothesis that an MCI with diagnostic characteristics of DLB would be associated with a worse prognosis than MCI-AD and suggest that different clinical presentations of MCI-LB may also be associated with different rates of clinical progression.

These results mirror recent findings on the prognosis of DLB or AD and in their cognitive prodromes: it appears that in both MCI and dementia,^20^ the presence of DLB-specific diagnostic characteristics is associated with a worse prognosis. DLB is associated with increased hospitalization,^1^ shorter time to full-time care, shorter survival time,^2^ and worse quality of life for patients and caregivers compared to AD.^21^ In a subset of this cohort, we have also previously found that those with MCI-LB were more likely to feature a progressive decline in cognitive scores than patients with MCI-AD.^3^ Our results here indicate that this also manifests in a greater annual risk of developing dementia than MCI-AD.

The specific association between cognitive fluctuations and visual hallucinations and a poorer prognosis in MCI may indicate that these are symptomatic of a more aggressive clinical phenotype. Both fluctuations^22^ and visual hallucinations^23^ are hypothesized to reflect particular patterns of neurodegeneration within Lewy body disease that may share a link,^24^ that is, the prominent cholinergic deficiency that may be more typical of DLB^25^ due to denervation of the basal forebrain^26^ and that may already be present at the MCI stage.^27^ Over the short term, people with Parkinson disease will not necessarily experience cognitive decline or progress to dementia,^28^ and among those who do, cholinergic denervation is more common,^29^ which is in line with our findings that parkinsonism and associated dopaminergic imaging findings may not be associated with a particularly increased risk of dementia within an MCI group, at least within the time frame of this study. Similarly while RBD may often lead to DLB, it may take several years to develop,^5^ consistent with the presence of RBD not being associated with increased risk of dementia in the short term as observed here. Drawing from a prospective cohort with in-depth and repeated assessment by an experienced clinical panel, this study has been able to characterize the dynamic clinical progression of MCI to dementia and thus provides clear evidence of the risks associated with specific clinical symptoms that may be identified in an MCI syndrome.

We previously identified that visual hallucinations were associated with a poorer cognitive prognosis in a subset of this cohort (n = 70), but this association was not observed with fluctuating cognition^3^; the daily variation in cognitive function characteristic of this symptom may obscure progressive decline in cognitive measures but still manifest in more clinically relevant functional declines as seen here (i.e., someone may score better on the day of a research cognitive assessment but at home show increased reliance on caregivers as a direct consequence of the intermittent but significant “lows” associated with this clinical symptom).

While RBD and parkinsonism are recognized risk factors for dementia within an older population in general,^5,6^ these were not observed to be risk factors over the shorter term in this study relative to those with MCI but without these symptoms. In contrast to the general population, within the context of MCI, these symptoms alone may confer no greater risk of transition to dementia than in AD because both RBD and Parkinson disease may exist as isolated diagnoses without cognitive impairment for many years (a decade or longer in the case of RBD). Alternatively, any additional increased risk may manifest only over the longer term or may be too small or underpowered to translate into meaningful effects without considering the broader clinical picture (e.g., when RBD or parkinsonism is present alongside other symptoms of DLB, as described in the first model), because DLB diagnostic characteristics in general appear to be associated with increased risk of dementia onset.

Current consensus criteria^12^ are such that MCI cases without apparent Lewy body, frontotemporal, vascular, or other differential diagnostic features meet criteria for a diagnosis of MCI-AD, and thus, these cases are described as such here. Despite the detailed and repeated assessment, it also remains possible that a number of these MCI cases may reflect nonneurodegenerative etiologies, which would not be expected to convert to dementia.^30^

These clinical diagnoses are limited by an absence of biomarkers specific to AD and consequent uncertainty in the association between observed features such as cognitive fluctuations or visual hallucinations and underlying Lewy body disease. While a subset of those who died underwent autopsy, to date, only 5 have entered our brain bank. Two with probable MCI-LB, both had neocortical Lewy body disease, and 3 diagnosed with MCI-AD all met the pathology criteria for AD, including being in Braak stages 5 and 6. While numbers are limited, this provides some gold standard validation for our diagnoses. Ultimately, fuller retrospective study of patients with neuropathologically confirmed Lewy body disease and/or AD could provide clearer evidence for the disease-specific (rather than symptom-specific) associations with dementia onset.

MIBG imaging was undertaken in only a subset of the full cohort; while this was not found to be a predictor of decline in this subgroup, our ability to draw broader conclusions for this measure is limited because this was not available for all, and it remains possible that those who did not undergo MIBG imaging could have a less accurate clinical characterization. In addition, the presence of RBD was judged from the clinical interview, with polysomnography not available; while the use of clinical interview is strongly supported (sensitivity 98%, specificity 74%^31^) in assessing RBD for DLB and MCI-LB diagnoses,^4,14^ it remains possible that an unknown number of this subgroup could be experiencing a non-REM parasomnia that would not be indicative of a synucleinopathy being present.

Overall mortality was low, with only 7 deaths by the time of data locking for this analysis, reflecting the early stage of disease in this cohort. The CIs around the effect sizes of predictors of death were therefore wide and the magnitude of these effects is more uncertain in contrast to the predictors of dementia. This may also account for the lack of observed associations with other expected covariates such as sex or local deprivation.

Diagnostic predictors (both biomarkers and clinical features) were included as binary absent/present variables in this analysis. Continuous quantifications of both biomarkers and clinical features may be more sensitive to mild differences in these and to any longitudinal change (e.g., worsening FP-CIT abnormalities over time) that may anticipate dementia onset.

Individuals with probable MCI-LB had slightly greater daily functional impairment than those with MCI-AD at baseline, although all had only mild impairment as reflected in their MCI diagnoses. Functional impairments were associated with motor, but not cognitive, impairments at baseline, suggesting that these reflect other barriers to independence in Lewy body disease. Individuals with probable MCI-LB were also more likely to be in receipt of cholinesterase inhibitors at baseline despite comparable cognitive function, consistent with recommendations and their local use in treating neuropsychiatric symptoms of Lewy body disease.^19^ Due to the observational nature of this study and collinearity with clinical variables, we were limited in our ability to assess the influences of cholinesterase inhibitors in dementia onset; addressing this research question would naturally require a randomized study with larger numbers.

An individual with MCI with clinical characteristics of DLB has a worse prognosis than an individual with MCI-AD, with increased annual risk of clinical conversion to dementia. The presence of cognitive fluctuations or visual hallucinations in particular is associated with worse prognosis. There may therefore be value in seeking information on the presence or absence of diagnostic features of Lewy body disease in MCI to aid the prospective identification of those at risk of further clinical decline.

## Glossary

AD: Alzheimer disease
AIC: Akaike information criterion
CI: confidence interval
DLB: dementia with Lewy bodies
FP-CIT: ^123^I-*N*-fluoropropyl-2β-carbomethoxy-3β-(4-iodophenyl) single-photon emission CT
HR: hazard ratio
LB: Lewy bodies
MCI: mild cognitive impairment
MIBG: ^123^I-metaiodobenzylguanidine
NIA-AA: National Institute on Aging–Alzheimer's Association
RBD: REM sleep behavior disorder

## References

[1] Mueller C, Perera G, Rajkumar AP, et al. Hospitalization in people with dementia with Lewy bodies: frequency, duration, and cost implications. Alzheimer's Demen Diagn Assess Dis Monit. 2018;10:143-152. 10.1016/j.dadm.2017.12.001 PMC5956805 29780862

[2] Mueller C, Soysal P, Rongve A, et al. Survival time and differences between dementia with Lewy bodies and Alzheimer's disease following diagnosis: a meta-analysis of longitudinal studies. Ageing Res Rev. 2019;50:72-80. 30625375 10.1016/j.arr.2019.01.005

[3] Hamilton CA, Matthews FE, Donaghy PC, et al. Prospective predictors of decline versus stability in mild cognitive impairment with Lewy bodies or Alzheimer's disease. Psychol Med. 2021;29(3):272-284. 10.1017/S0033291720001130 32366348

[4] McKeith IG, Ferman TJ, Thomas AJ, et al. Research criteria for the diagnosis of prodromal dementia with Lewy bodies. Neurology. 2020;94:1-13. 10.1212/WNL.0000000000009323 PMC7274845 32241955

[5] Claassen DO, Josephs KA, Ahlskog JE, Silber MH, Tippmann-Peikert M, Boeve BF. REM sleep behavior disorder preceding other aspects of synucleinopathies by up to half a century. Neurology. 2010;75:494-499. 20668263 10.1212/WNL.0b013e3181ec7fac PMC2918473

[6] Aarsland D. Cognitive impairment in Parkinson's disease and dementia with Lewy bodies. Parkinson Relat Disord. 2016;22:S144-S148. 10.1016/j.parkreldis.2015.09.034 26411499

[7] Mauri M, Sinforiani E, Zucchella C, Cuzzoni MG, Bono G. Progression to dementia in a population with amnestic mild cognitive impairment: clinical variables associated with conversion. Funct Neurol. 2012;27:49-54. 22687167 PMC3812753

[8] Scarmeas N, Brandt J, Albert M, et al. Delusions and hallucinations are associated with worse outcome in Alzheimer disease. Arch Neurol. 2005;62:1601-1608. 16216946 10.1001/archneur.62.10.1601 PMC3028538

[9] D'Antonio F, Reeves S, Sheng Y, et al. Misidentification subtype of Alzheimer's disease psychosis predicts a faster cognitive decline. CPT Pharmacometrics Syst Pharmacol. 2019;8:308-315. 30779330 10.1002/psp4.12389 PMC6533361

[10] Donaghy PC, Taylor J-P, O'Brien JT, et al. Neuropsychiatric symptoms and cognitive profile in mild cognitive impairment with Lewy bodies. Psychol Med. 2018;48:2384-2390. 29362011 10.1017/S0033291717003956

[11] Schumacher J, Taylor J-P, Hamilton CA, et al. Quantitative EEG as a biomarker in mild cognitive impairment with Lewy bodies. Alzheimers Res Ther. 2020;12:82. 32641111 10.1186/s13195-020-00650-1 PMC7346501

[12] Albert MS, DeKosky ST, Dickson D, et al. The diagnosis of mild cognitive impairment due to Alzheimer's disease: recommendations from the National Institute on Aging-Alzheimer's Association workgroups on diagnostic guidelines for Alzheimer's disease. Alzheimers Dement. 2011;7:270-279. 21514249 10.1016/j.jalz.2011.03.008 PMC3312027

[13] McKhann GM, Knopman DS, Chertkow H, et al. The diagnosis of dementia due to Alzheimer's disease: recommendations from the National Institute on Aging-Alzheimer's Association workgroups on diagnostic guidelines for Alzheimer's disease. Alzheimers Dement. 2011;7:263-269. 21514250 10.1016/j.jalz.2011.03.005 PMC3312024

[14] McKeith IG, Boeve BF, Dickson DW, et al. Diagnosis and management of dementia with Lewy bodies: fourth consensus report of the DLB Consortium. Neurology. 2017;89:88-100. 28592453 10.1212/WNL.0000000000004058 PMC5496518

[15] Thomas AJ, Donaghy PC, Roberts G, et al. Diagnostic accuracy of dopaminergic imaging in prodromal dementia with Lewy bodies. Psychol Med. 2019;49:396-402. 29692275 10.1017/S0033291718000995 PMC6331684

[16] Xue H, Sun Q, Liu L, et al. Risk factors of transition from mild cognitive impairment to Alzheimer's disease and death: a cohort study. Compr Psychiatry. 2017;78:91-97. 28806610 10.1016/j.comppsych.2017.07.003

[17] Department for Communities and Local Government. The English Indices of Deprivation 2010. Department for Communities and Local Government London; 2011.

[18] Jackson CH. Multi-state models for panel data: the msm package for R. J Stat Softw. 2011;38:1-29.

[19] Taylor J-P, McKeith IG, Burn DJ, et al. New evidence on the management of Lewy body dementia. Lancet Neurol. 2020;19:157-169. 31519472 10.1016/S1474-4422(19)30153-X PMC7017451

[20] Price A, Farooq R, Yuan J-M, Menon VB, Cardinal RN, O'Brien JT. Mortality in dementia with Lewy bodies compared with Alzheimer's dementia: a retrospective naturalistic cohort study. BMJ Open. 2017;7:e017504. 10.1136/bmjopen-2017-017504 PMC5695389 29101136

[21] Wu YT, Clare L, Hindle JV, Nelis SM, Martyr A, Matthews FE. Dementia subtype and living well: results from the Improving the experience of Dementia and Enhancing Active Life (IDEAL) study. BMC Med. 2018;16:140. 30200957 10.1186/s12916-018-1135-2 PMC6131832

[22] O'Dowd S, Schumacher J, Burn DJ, et al. Fluctuating cognition in the Lewy body dementias. Brain. 2019;142:3338-3350. 31411317 10.1093/brain/awz235

[23] Erskine D, Taylor JP, Thomas A, et al. Pathological changes to the subcortical visual system and its relationship to visual hallucinations in dementia with Lewy bodies. Neurosci Bull. 2019;35:295-300. 30729454 10.1007/s12264-019-00341-4 PMC6426914

[24] O'Brien JT, Firbank MJ, Mosimann UP, Burn DJ, McKeith IG. Change in perfusion, hallucinations and fluctuations in consciousness in dementia with Lewy bodies. Psychiatry Res. 2005;139:79-88. 15964748 10.1016/j.pscychresns.2005.04.002

[25] Lemstra AW, Eikelenboom P, van Gool WA. The cholinergic deficiency syndrome and its therapeutic implications. Gerontology. 2003;49:55-60. 12457052 10.1159/000066508

[26] Colloby SJ, Elder GJ, Rabee R, O'Brien JT, Taylor JP. Structural grey matter changes in the substantia innominata in Alzheimer's disease and dementia with Lewy bodies: a DARTEL-VBM study. Int J Geriatr Psychiatry. 2017;32:615-623. 27197956 10.1002/gps.4500 PMC5434823

[27] Schumacher J, Thomas AJ, Peraza LR, et al. EEG alpha reactivity and cholinergic system integrity in Lewy body dementia and Alzheimer's disease. Alzheimers Res Ther. 2020;12:46. 32321573 10.1186/s13195-020-00613-6 PMC7178985

[28] Williams-Gray CH, Foltynie T, Brayne CE, Robbins TW, Barker RA. Evolution of cognitive dysfunction in an incident Parkinson's disease cohort. Brain. 2007;130:1787-1798. 17535834 10.1093/brain/awm111

[29] Ray NJ, Bradburn S, Murgatroyd C, et al. In vivo cholinergic basal forebrain atrophy predicts cognitive decline in de novo Parkinson's disease. Brain. 2018;141:165-176. 29228203 10.1093/brain/awx310 PMC5837422

[30] McWhirter L, Ritchie C, Stone J, Carson A. Functional cognitive disorders: a systematic review. Lancet Psychiatry. 2019;7(2):191-207. 31732482 10.1016/S2215-0366(19)30405-5

[31] Boeve BF, Molano JR, Ferman TJ, et al. Validation of the Mayo Sleep Questionnaire to screen for REM sleep behavior disorder in an aging and dementia cohort. Sleep Med. 2011;12:445-453. 21349763 10.1016/j.sleep.2010.12.009 PMC3083495

